# Precipitation and Solubility of Calcium Hydrogenurate Hexahydrate

**DOI:** 10.6028/jres.097.015

**Published:** 1992

**Authors:** V. Babić-Ivančić, H. Füredi-Milhofer, N. Brničević, M. Marković

**Affiliations:** Rudjer Bošković Institute, Zagreb, Croatia; Rudjer Bošković Institute and Faculty of Science, University of Zagreb, Zagreb, Croatia and American Dental Association Health Foundation, National Institute of Standards and Technology, Gaithersburg, MD 20899

**Keywords:** calcium hydrogenurate hexahydrate, identification, precipitation, solubility, solubility product, urinary stone formation

## Abstract

Solid phases formed in the quaternary system: uric acid—calcium hydroxide —hydrochloric acid—water aged for 2 months at 310 K were studied to determine conditions for calcium hydrogenurate hexahydrate, Ca(C_5_H_3_N_4_O)_2_ · 6H_2_O precipitation. The precipitates were identified by chemical and thermogravimetric analyses, x-ray powder diffraction, infrared spectroscopy, light microscopy, and scanning electron microscopy. In the precipitation diagram the concentration region in which calcium hydrogenurate hexahydrate precipitated as a single solid phase was established.

The solubility of calcium hydrogenurate hexahydrate was investigated in the pH range from 6.2 to 10.1 at different temperatures. The total soluble and ionic concentration of calcium (atomic absorption spectroscopy and Ca-selective electrode), total urate concentration (spectrophotometry), and pH were determined in equilibrated solutions. The data are presented in the form of tables and chemical potential diagrams. By using these data the thermodynamic solubility products of calcium hydrogenurate hexahydrate, *K*_s_ = *a*(Ca^2+^) · *a*^2^(C_5_H_3_N_4_O_3_^−^), were determined:

$$\begin{array}{lll} pK_{s} & =\;10.12\pm 0.07 & \text{at}\;288\;\text{K,}\\ pK_{s} & =\;9.81\pm 0.09 & \text{at}\;298\;\text{K,}\\ pK_{s} & =\;9.28\pm 0.04 & \text{at}\;310\;\text{K,}\;\text{and}\\ pK_{s} & =\;9.01\pm 0.03 & \text{at}\;318\;K\text{.} \end{array}$$

The formation of calcium hydrogenurate hexahydrate crystals in urinary tract of patients with pathologically high concentrations of calcium and urates (hypercalciuria and hyperuricosiuria) is possible.

## 1. Introduction

The formation of urinary stones of various composition (urolithiasis) is a painful and crippling pathological process in the urinary tract. The occurrence of stones containing pure uric acid, C_5_H_4_N_4_O_3_, H_2_U, has been reported [1] but usually urate calculi have a layer-like composition consisting of additional crystalline compounds, i.e., calcium oxalates, calcium phosphates, cystine, etc. [1,2]. It has been proposed that uric acid and/or sodium hydrogenurate may serve as templates for formation of calcium salts in renal stones [3–6]. Calcium urates have also been considered as possible constituents of urinary stones [7–9] but there is no evidence about them as separate solid phases. Gouty arthritis is another disease in which urates form pathological deposits [10].

Because of their physiological relevance, the precipitation, dissolution, and solubility of anhydrous uric acid [11–14], uric acid dihydrate [14,15] and sodium hydrogenurate monohydrate [11,16–18] have been studied in detail. There is, however, a limited number of investigations on calcium urates. Only the thermodynamically stable calcium hydrogenurate hexahydrate, Ca(C_5_H_3_N_4_O_3_)_2_ · 6H_2_O, Ca(HU)_2_ · 6H_2_O, has been prepared and characterized [19, 20] but no solubility data of this compound have as yet been reported.

In this paper we describe the formation of Ca(HU)_2_ · 6H_2_O in the four component system: uric acid—calcium hydroxide—hydrochloric acid—water at physiological temperature (310 K). The solubility products of Ca(HU)_2_ · 6H_2_O at 288, 298, 310, and 318 K are reported. These results should facilitate understanding of the possible precipitation of Ca(HU)_2_ · 6H_2_O in a physiological environment.

## 2. Methods

### 2.1 Preparation and Identification

Calcium hydrogenurate hexahydrate was prepared by equilibrating commercial uric acid (Sigma Chemical^2^) with aqueous solutions of calcium hydroxide to which hydrochloric acid was added when neccessary to adjust the pH. To ensure slow crystallization and recrystallization the samples were kept without agitation for several weeks in a nitrogen atmosphere. After completion of the reaction, crystals were examined in solution by an inverted light microscope (Leitz, Wetzlar), subsequently filtered and characterized by physicochemical and analytical methods. X-ray diffraction (XRD) patterns (Phillips x-ray diffractometer with proportional counter, using graphite monochromated Cu*K*α radiation) were taken from moist precipitates and after extended air-drying. Infrared spectra (4000 – 200 cm^−1^) were recorded as nujol mulls (Perkin Elmer, Model 580B spectrophotometer). Scanning electron micrographs (Cambridge Stereo Scan 600) were obtained from dried crystals. In some samples the chemical composition was confirmed. Calcium was determined by atomic absorption spectroscopy (Jarel Ash) and gravimetry; carbon, hydrogen, and nitrogen by microanalysis, and content of water and purine by thermogravimetric analysis (Cahn RG electromicroanalytical balance, heating rate 2 °C/min in air).

### 2.2 Solubility Determinations

Solubility determinations of Ca(HU)_2_ · 6H_2_O were carried out (a) in the original supernatant after sample preparation and (b) in water and aqueous solutions of uric acid or calcium chloride.

- Freshly prepared Ca(HU)_2_ · 6H_2_O crystals were reequilibrated with their respective supernatants by shaking them for 24 h at 310 K.
- Dried crystals were equilibrated at different temperatures (288, 298, 310, and 318 K) in triply distilled water and in uric acid or calcium chloride solutions by shaking them in a thermostated water bath for 24 h (at 288 and 298 K) or 3 h (at 310 and 318 K).

After equilibration, the concentration of total calcium (atomic absorption), ionic calcium (Ca-selective electrode), total urate (direct spectrophotometric determination at 285 nm [14]), and pH were determined in all supernatants and the solid phase was identified by XRD.

### 2.3 Processing of Solubility Data

Computation of ion activities and solubility product of Ca(HU)_2_ · 6H_2_O,

$$K_{s}=a\left({\text{Ca}}^{2+}\right)\cdot a^{2}\left({\text{HU}}^{-}\right)$$ (1)

was made by an iterative procedure using the experimentally determined concentrations of all species in equilibrated solutions and literature values of uric acid [13,15,21] and water [22,23] dissociation constants for different temperatures (Table 1).

For calculating the activity coefficients, *y*, the Davies modification of the extended Debye-Hückel equation was used:

$$\log\;y=-Az^{2}\left(\sqrt{I}/\left(1+\sqrt{I}\right)-0.2I\right)$$ (2)

where *z* is valence charge of the ion and *A* is the Debye constant having the values of 0.5002,0.5115, 0.5242, and 0.5296 for 288, 298, 310, and 318 K, respectively [23,24]. Ionic strength, *I*, was defined as *I* = 0.5 Σ*cz*^2^ where *c* is the concentration of corresponding ionic species.

The solubility data were recalculated in terms of the ion activity products *a*(Ca^2+^) · *a*^2^(OH^−^) and *a*(H^+^) · *a*(HU^−^). The following treatment, based on considerations of the chemical potentials of the components in equilibrium with the solid phase [25,26] was then applied:

Multiplying both sides of Eq. (1) by *a*^2^(H^+^) · *a*^2^(OH^−^) yields

$${\left[a\left(H^{+}\right)\cdot a\left({\text{HU}}^{-}\right)\right]}^{2}\cdot \left[a\left({\text{Ca}}^{2+}\right)\cdot a^{2}\left({\text{OH}}^{-}\right)\right]=K_{s}\cdot {K_{w}}^{2}.$$ (3)

Taking logarithms and rearranging gives

$$\log\;a\left(H^{+}\right)\cdot a\left({\text{HU}}^{-}\right)=0.5\log\;a\left({\text{Ca}}^{2+}\right)\cdot a^{2}\left({\text{OH}}^{-}\right)+0.5\log\;K_{s}\cdot {K_{w}}^{2}.$$ (4)

Equation (4) describes a straight line whose slope is 0.5 if the composition of the solid phase is Ca(HU)_2_ · 6H_2_O.

## 3. Results and Discussion

### 3.1 Precipitation and Characterization of Ca(HU)_2_ · 6H_2_O

**Fig. 1:**
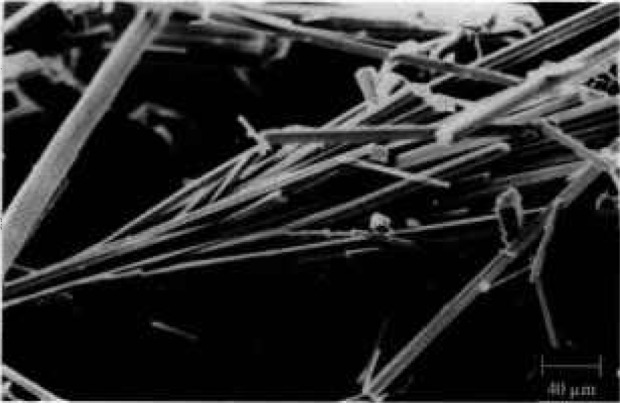
Scanning electron micrograph of calcium hydrogenurate hexahydrate crystals.

When calcium hydroxide solution was added to crystalline uric acid the latter dissolved but simultaneously Ca(HU)_2_ · 6H_2_O crystals started forming. After completion of the reaction the solid phase consisted of layered aggregates of large, elongated crystals as shown in Fig. 1. The XRD powder pattern of moist precipitates suggested the presence of some amorphous material in addition to the crystalline matter. During prolonged air-drying, however, further recrystallization occurred resulting in Ca(HU)_2_ · 6H_2_O crystals with a well resolved XRD powder pattern. The *d*-values and relative intensities obtained were in very good agreement with those already reported [19]. Infrared spectra confirmed the presence of HU^−^ and water molecules but comparison of the position of *ν*(CO) in the spectrum of Ca(HU)_2_ · 6H_2_O with the position of the same absorption in the spectra of Ca(HU)_2_L_3_ (L=dimethylsulphoxide or N,N-dimethylformamide) indicated that calcium atoms in the former compound could be coordinated by water molecules rather than by hydrogenurate ions; the presence of aquo cation [Ca(H_2_O)_6_^2+^] in the structure of Ca(HU)_2_ · 6H_2_O is anticipated [27]. Chemical and thermogravimetric analyses (TGA) revealed the presence of two purine rings and six water molecules per calcium atom (Table 2). TGA showed the loss of 22.2±0.5 mass % in the temperature range from 387 to 640 K corresponding to six water molecules and an additional 56.6 ±1.1 mass % loss up to 870 K, due to the decomposition of purine.

**Fig. 2:**
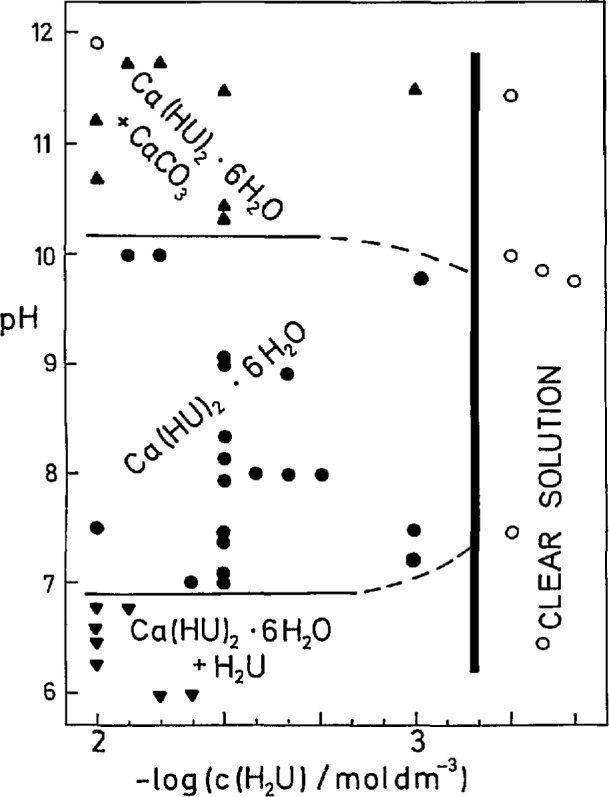
Precipitation diagram for the system: uric acid—calcium hydroxide—hydrochloric acid—water observed 2 months after sample preparation at 310 K. The data points indicate initial uric acid concentration, c(H_2_U), and pH determined in equilibrated solutions. In all systems initial c(Ca) = 1.5 · c(H_2_U). The precipitation boundary (thick line) separates clear solutions (o) from the region of precipitates. Phase boundaries (thin lines) separate the region of pure calcium hydrogenurate hexahydrate, Ca(HU)_2_ · 6H_2_O (●) from the regions of its mixture with CaCO_3_ (▲) and uric acid (▼).

The precipitation diagram of H_2_U-Ca(OH)_2_-HCI-H_2_O systems aged for 2 months at 310 K shows the composition of the prevailing solid phases in a wide range of equilibrium pH and total uric acid concentration (Fig. 2). In all samples the molar ratio *c*(Ca)/*c*(H_2_U) was 1.5. Ca(HU)_2_ · 6H_2_O crystallized as a single solid phase at *c*(H_2_U) > 1.1 mmol dm^−3^ and 7 < pH < 10. At pH < 7 some uric acid was always admixed while at pH>10 the coprecipitation of a small amount of CaCO_3_ could not be avoided even when the samples were prepared in a dry-box under a nitrogen atmosphere. From the precipitation boundary which is positioned at *c*(H_2_U) ~8 · 10^−4^ mol dm^−3^, an approximate value of the solubility product of Ca(HU)_2_ · 6H_2_O was calculated [Eq. (1)] to be of the order of magnitude of 10^−10^.

### 3.2 Solubility Products of Ca(HU)_2_ · 6H_2_O

The equilibration time for solubility determinations of Ca(HU)_2_ · 6H_2_O depended on temperature and pH. For systems at 310 K, pH>7 and for all systems at 288 and 298 K the equilibration time was 24 h. The systems at 310 K, pH < 7.5 and all systems at 318 K were equilibrated for 3 h to avoid precipitation of uric acid. Preliminary kinetic experiments monitoring the rate of dissolution of Ca(HU)_2_ · 6H_2_O into water showed that equilibrium is soon established, i.e., at 288 and 298 K consistent results were obtained between 3 and 24 h while at 310 and 318 K between 20 min and 3h.

**Fig. 3:**
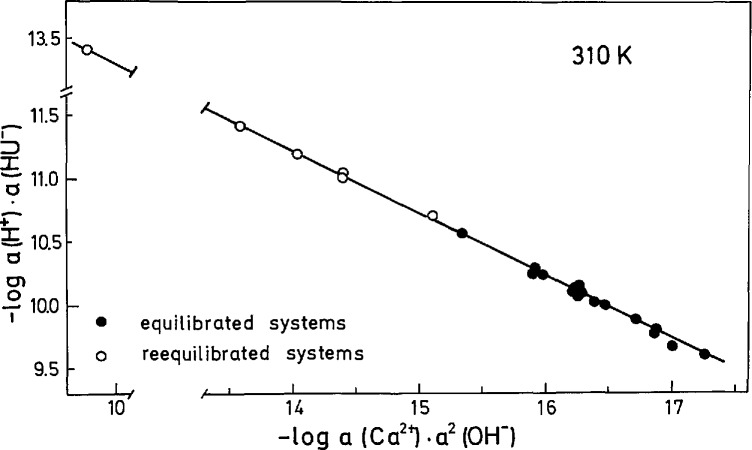
Potential diagram calculated by using the solubility data for Ca(HU)_2_ · 6H_2_O at 310 K (Table 3) and dissociation constants of H_2_U and H_2_O (Table 1). The slope of the straight line is 0.505.

In Table 3 are given the concentrations of soluble urate and calcium, pH, and calculated ionic strengths (a) in reequilibrated systems after 24 h (systems 1 to 6) and (b) in the systems after 3 h equilibration of Ca(HU)_2_ · 6H_2_O in water (systems 7 to 13), uric acid (systems 14 to 18) and calcium chloride solutions (systems 19 to 22) at the physiological temperature of 310 K. No significant difference between the concentration of total soluble calcium and ionic calcium was detected. The values were in the range of experimental error (±2.8%) indicating that Ca^2+^ is the dominant calcium species in urate solutions and that calcium does not form strong soluble complexes with urate anions. The mean value of total soluble and ionic calcium concentration is taken as equilibrium concentrations, *c* (Ca)_eq_. Calculated thermodynamic solubility products, *K*_S_(*I* =0), listed in Table 3 have an average value of (5.3±0.4) · 10^−10^. The experimental data obtained at 310 K in reequilibrated (Fig. 3, open circles) and equilibrated systems (Fig. 3, filled circles) are plotted in the form of a chemical potential plot [Eq. (4)] giving a straight line with the slope of 0.505 indicating that in the range of 6.6<pH<10.1 the solid phase in equilibria with supernatant was Ca(HU)_2_ · 6H_2_O.

**Fig. 4:**
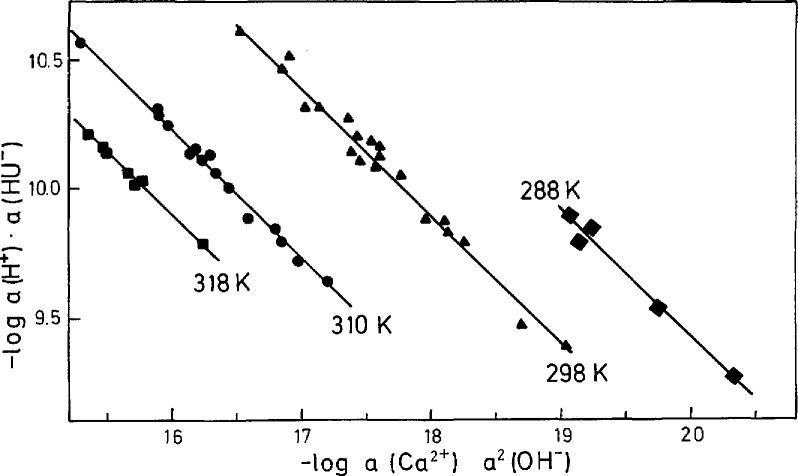
Potential diagram calculated by using the solubility data for Ca(HU)_2_ · 6H_2_O (Table 4) and dissociation constants of H_2_U and H_2_0 (Table 1). The slopes of the straight lines are 0.483 (288 K), 0.510 (298 K), and 0.492 (318 K). The results determined in equilibrated systems at 310 K are given for comparison (slope of 0.505).

In Table 4 are listed the concentrations of all components in solutions equilibrated with Ca(HU)_2_ · 6H_2_O at 288 K (systems 23 to 27), 298 K (systems 28 to 47) and 318 K (systems 48 to 54). Ca(HU)_2_ · 6H_2_O was equilibrated in water (systems 23 to 25, 28 to 33, and 48 to 50), uric acid solutions (systems 26 and 27, 34 to 39, and 51 to 54) or calcium chloride solutions (systems 40 to 48). The *K*_s_(*I* = 0) values obtained show differences in the range of experimental error giving an average value of (7.6±l.l) · 10^−11^ at 288 K, (1.6+0.3) · 10^−10^ at 298 K, and (9.8 ± 0.6) · 10^−10^ at 318 K. Chemical potential plots, showing linear dependence of −log *a*(H^+^) · *a*(HU^−^) vs −log *a*(Ca^2+^) · *a*^2^(OH^−^) with slopes of 0.483, 0.510, and 0.492 for 288, 298, and 318 K, respectively (Fig. 4), confirmed that the solid phase has a molar Ca/urate ratio of 1:2. The data from equilibrated systems at 310 K (Fig. 3, filled circles) are shown in Fig. 4 for comparison. Solubility of Ca(HU)_2_ · 6H_2_O increases with increasing temperature.

The solubility products can be determined from the intercepts of the straight lines in the chemical potential plots [Eq. (4), Fig. 4] but with less precision than by direct computation from equilibrium concentrations. Specifically, in the pH range from 6.5 to 8.5 the dominant urate species is HU^−^ and the calculated values of *a*(HU^−^) are relatively insensitive to small uncertainties in pH. On the other hand in the potential diagram (Fig. 4), the small changes in pH alter slopes of the straight lines and cause significant differences in intercepts and corresponding *K*_s_ values.

### 3.3 Possible Formation of Ca(HU)_2_ · 6H_2_O in Urines

From the precipitation diagram shown in Fig. 2 it is possible to conclude that in the physiological range of urinary pH (5.0<pH<6.5) coprecipitation of uric acid and Ca(HU)_2_ · 6H_2_O might be expected while in slightly alkaline urines Ca(HU)_2_ · 6H_2_O could precipitate as a single solid phase. In the physiological range of total urinary urate and calcium concentrations, 1 < *c* (U)_tot_ < 3 mmol dm^−3^ and l<*c*(Ca)_tot_<4 mmol dm^−3^ (*c*(Ca^2+^)≈0.5 · *c*(Ca)_tot_, *I* = 0.3 mol dm^−3^, 310 K) the ion activity product, *IAP*=*a*(Ca^2+^) · *a*^2^(HU^−^), varies from 7 · 10^−12^ to 2 · 10^−9^. Supersaturation with respect to precipitation of Ca(HU)_2_ · 6H_2_O (*S=IAP/K*_s_) varies from 0.01 to 3.8. It is obvious that these urines are undersaturated or slightly supersaturated with respect to precipitation of Ca(HU)_2_ · 6H_2_O and spontaneous precipitation of this salt would not be expected. At higher supersaturations, eventually, coprecipitation or overgrowth of Ca(HU)_2_. 6H_2_O on previously formed uric acid crystals may be possible. The conditions for precipitation of Ca(HU)_2_ · 6H_2_O exsist in urines of patients with both hyperuricosiuria and hypercalciuria.

## 4. Conclusions

The thermodynamic solubility products, *K*_s_, of Ca(HU)_2_-6H_2_O have the values of (7.6 ±1.1) · 10^−11^ at 288 K, (1.6 + 0.3) · 10^−10^ at 298 K, (5.3±0.4) · 10^−10^ at 310 K, and (9.8 + 0.6) · 10^−10^ at 318 K. In the physiological range of urinary concentrations of urate and calcium, and pH, spontaneous precipitation of Ca(HU)_2_ · 6H_2_O would not be expected, while in urines of patients with high concentrations of constituent ions (pathological conditions) the formation of Ca(HU)_2_ · 6H_2_O is possible.

## Figures and Tables

**Table 1:** Dissociation constants of uric acid and water at different temperatures

Equilibria	p*K*				Ref.
	(288 K)	(298 K)	(310 K)	(318 K)	
1. H_2_U⇄H^+^+HU^−^	5.65	5.61	5.44	5.34	[13,15]
2. HU^−^⇄H^+^+U^2−^	9.15	9.15	10.51	10.51	[21]
3. H_2_O⇄H^+^+OH^−^	14.35	13.99	13.61	13.39	[22,23]

**Table 2:** Preparative conditions and thermogravimetric and chemical analysis of Ca(HU)_2_ · 6H_2_O (s)

c(H_2_U)_init_ (mmol dm^−3^)	c(Ca)_init_ (mmol dm^−3^)	pH_init_	TG analysis (%)		Ca	Chemical analysis (%)		N
			H_2_O	purine		C	H	
10.0	15.0	7.30	22.07	55.28	8.20	24.92	3.65	23.86
4.0	4.4	7.40	21.23	56.70		24.77	4.00	25.01
10.0	15.0	7.50	22.43	57.94	8.16	24.96	3.56	24.66

		calculated values:	22.41	56.88	8.30	24.80	3.76	23.23

**Table 3:** Experimental solubility data and calculated *K*_s_ values for Ca(HU)_2_ · 6H_2_O (s) at 310 K

System No.^a^	*c*(U)_eq_ (mmol dm^−3^)	*c*(Ca)_eq_ (mmol dm^−3^)	pH_eq_	*I*_eq_ (mmol dm^−3^)	*K*_s_ · 10^10^(*I* = 0)
1	0.741	3.010	10.06	8.60	4.15
2	0.609	2.800	7.95	8.00	5.75
3	0.605	2.970	8.17	8.40	5.93
4	0.563	2.900	7.40	8.40	4.98
5	0.577	2.860	7.76	8.30	5.23
6	0.551	2.890	7.76	8.30	4.81
7	1.110	0.566	7.15	1.68	5.07
8	1.160	0.594	7.14	1.76	5.77
9	1.140	0.609	7.09	1.78	5.68
10	1.160	0.609	6.83	1.78	5.68
11	1.120	0.563	7.16	1.70	5.22
12	1.110	0.583	7.15	1.72	5.20
13	1.120	0.567	7.27	1.69	5.21
14	1.150	0.569	6.65	1.68	5.06
15	1.160	0.592	6.79	1.75	5.49
16	1.190	0.594	6.93	1.77	5.92
17	1.170	0.586	6.87	1.74	5.61
18	1.130	0.548	7.06	1.65	5.05
19	0.246	50.000	6.83	150.10	5.22
20	0.378	10.200	6.92	30.60	5.01
21	0.484	5.280	6.91	15.80	5.37
22	0.773	1.380	6.96	4.14	5.08

a Systems 1 to 6 were reequilibrated in original supernatants for 24 h. In systems 7 to 13, Ca(HU)_2_ · 6H_2_O was equilibrated in water. In systems 14 to 18 the initial uric acid concentrations were 0.1, 0.1, 0.08, 0.05, and 0.03 mmol dm^−3^, respectively. In systems 19 to 22 the initial calcium chloride concentrations were 50, 10, 5, and 1 mmol dm^−3^, respectively. Equilibration time for systems 7 to 22 was 3 h.

**Table 4:** Experimental solubility data and calculated *K*_s_ values for Ca(HU)_2_ · 6H_2_O (s) at different temperatures (288, 298, and 318 K)

System No.^a^	*T/K*	*c*(U)_eq_ (mmol dm^−3^)	*c*(Ca)_eq_ (mmol dm^−3^)	pH_eq_	*I*_eq_ (mmol dm^−3^)	*K*_s_ · 10^10^(*I*=0)
23	288	0.631	0.323	6.53	0.97	0.87
24		0.631	0.328	6.61	0.95	0.86
25		0.579	0.285	6.61	0.84	0.64
26		0.736	0.336	5.93	0.92	0.65
27		0.675	0.312	6.26	0.90	0.76

28	298	0.776	0.393	6.74	0.12	1.62
29		0.736	0.387	6.88	1.13	1.49
30		0.677	0.386	6.96	1.11	1.28
31		0.781	0.391	6.95	1.17	1.71
32		0.727	0.347	6.95	1.05	1.33
33		0.721	0.384	6.68	1.11	1.35
34		0.819	0.397	6.20	1.13	1.36
35		0.935	0.414	6.38	1.23	2.10
36		0.820	0.377	6.69	1.14	1.72
37		0.886	0.391	7.04	1.22	2.22
38		0.777	0.346	7.04	1.08	1.53
39		0.731	0.396	6.60	1.13	1.39
40		0.119	50.000	6.43	150.10	1.08
41		0.115	50.000	6.97	150.10	1.15
42		0.228	10.000	6.72	30.11	1.70
43		0.226	10.000	6.87	30.11	1.72
44		0.239	5.120	6.56	15.35	1.14
45		0.490	1.000	6.98	3.25	1.56
46		0.499	1.230	6.98	3.71	1.87
47		0.496	1.270	6.76	3.78	1.82

48	318	1.460	0.678	7.29	1.99	9.62
49		1.360	0.719	7.29	2.12	9.47
50		1.420	0.703	7.34	2.10	10.20
51		1.440	0.700	6.90	2.11	10.04
52		1.430	0.624	7.16	1.99	9.12
53		1.360	0.715	7.18	2.11	9.37
54		1.480	0.705	7.15	2.15	10.89

a In systems 23 to 25, 28 to 33, and 48 to 50, Ca(HU)_2_ · 6H_2_O was equilibrated in water. In systems 26, 34 to 38, 51 and 54 initial uric acid concentration was 0.1 mmol dm^−3^ and in systems 27, 39, 52, and 53 it was 0.05 mmol dm^−3^. In systems 40 to 47 initial calcium chloride concentrations were 50,50,10,10, 5,1,1, and 1 mmol dm^−3^, respectively. Equilibration time for systems 23 to 47 was 24 h and for systems 48 to 54, 3 h.

## Footnotes

2 Certain commercial materials and equipment are identified in this paper to specify the experimental procedure. In no instance does such identification imply recommendation or endorsement by the National Institute of Standards and Technology or the ADA Health Foundation or that the material or equipment identified is necessarily the best available for the purpose.
